# Supplementary figures and images for: Characterization of intracellular calcium mobilization induced by remimazolam, a newly approved intravenous anesthetic
Source: PLoS One. 2022 Feb 1;17(2):e0263395. doi: 10.1371/journal.pone.0263395 (PMC8806057; doi:10.1371/journal.pone.0263395)

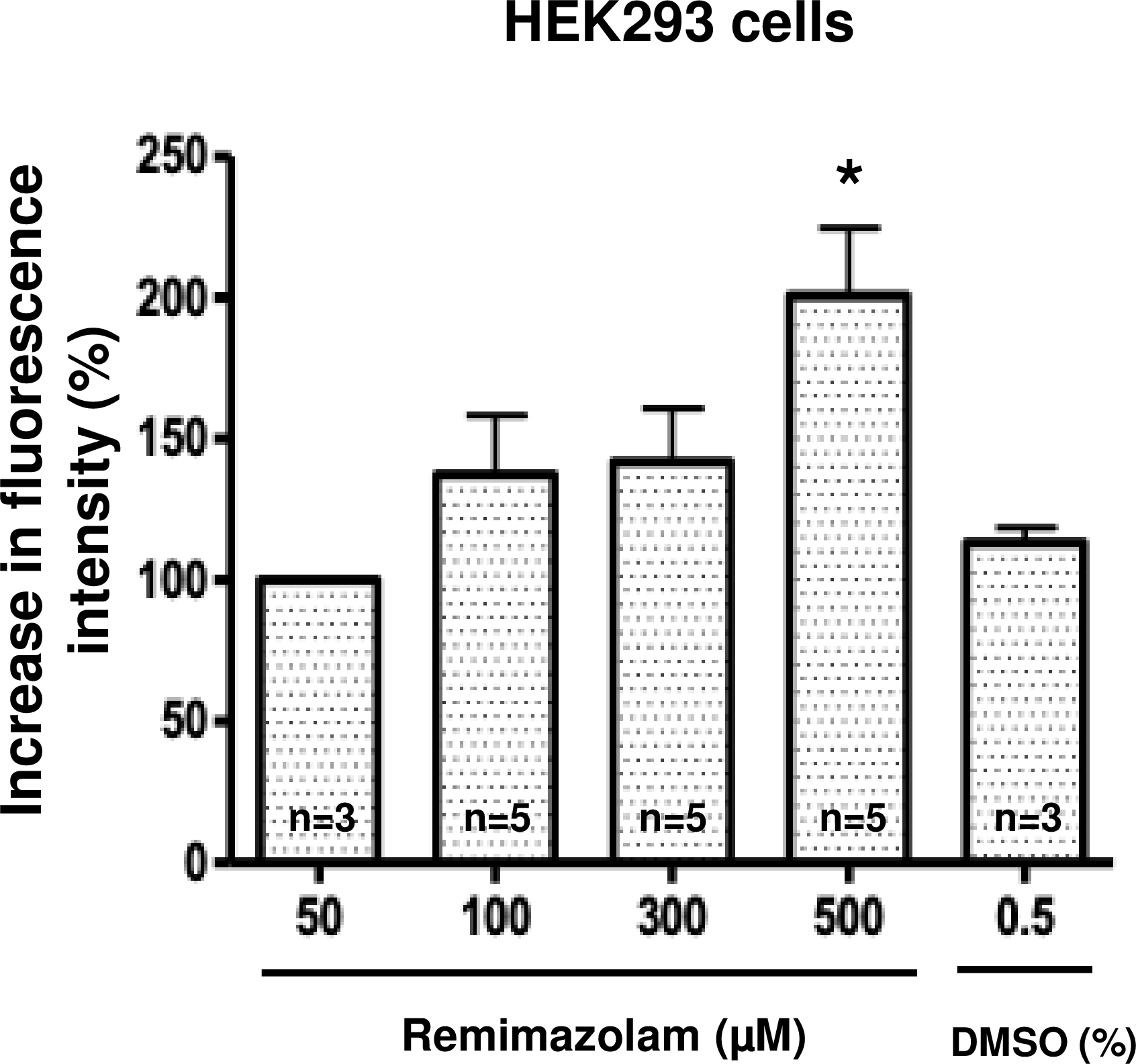

Supplement: S1 Fig — (A) Remimazolam at a concentration greater than or equal to 300 μM significantly induced the elevation of intracellular calcium in a dose-dependent manner in HEK293 cells. (n = 3–5, * p < 0.05, compared to control, one-way ANOVA followed by Dunnett’s post-test). (TIF) [file pone.0263395.s001.tif]

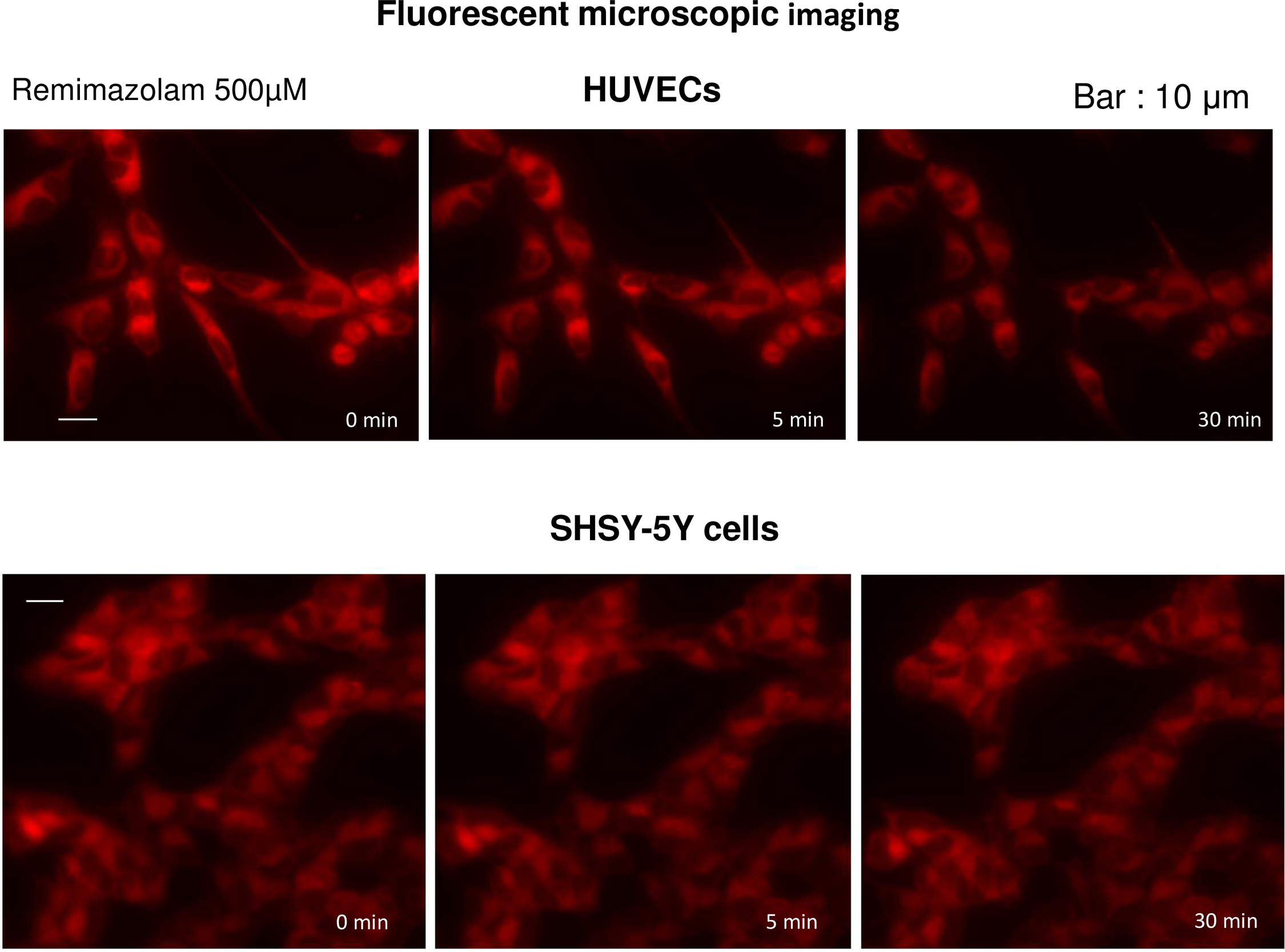

Supplement: S2 Fig — ER-tracker was capable of staining ER in living HUVECs and SHSY-5Y cells, remimazolam did not elicit the morphological changes of ER. (above: HUVECs, below: SHSY-5Y cells). (TIF) [file pone.0263395.s002.tif]

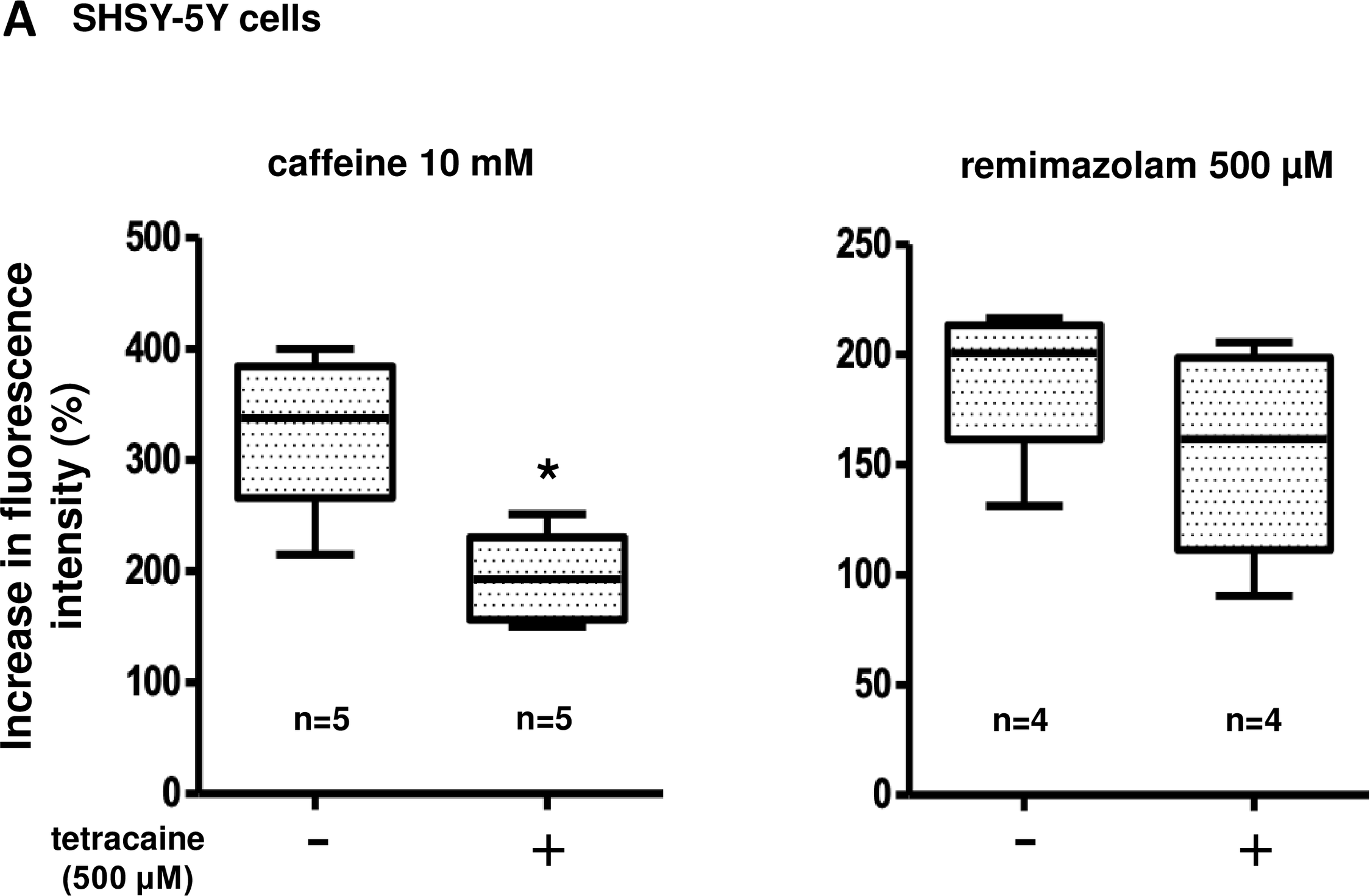

Supplement: S3 Fig — Caffeine, a ryanodine receptor (RYR) agonist, at 10 mM induced the intracellular calcium in SHSY-5Y cells. Fifteen minutes-pretreatment with tetracaine, a RYR antagonist, at 500 μM significantly inhibited the caffeine-induced calcium mobilization (n = 5, * p = 0.0159, compared to control, Mann Whitney test), however, 15 minutes-pretreatment with tetracaine at 500 μM did not significantly influence the 300 μM remimazolam-induced calcium elevation. Data represent the mean ± SEM (n = 4, p = 0.3429, compared to control, Mann Whitney test). (TIF) [file pone.0263395.s003.tif]
