# Supplementary material for: Characterization of intracellular calcium mobilization induced by remimazolam, a newly approved intravenous anesthetic
Source: PLoS One. 2022 Feb 1;17(2):e0263395. doi: 10.1371/journal.pone.0263395 (PMC8806057; doi:10.1371/journal.pone.0263395)
Supplement: Attachment — Submitted filename: GENERAL COMMENTS TO THE REVIEWER #1.docx [file pone.0263395.s005.docx]

**GENERAL COMMENTS TO THE REVIEWER #1**

**RESPONSES TO THE REVIEWER #1’s COMMENTS:**

1. COMMENT: The authors have done much to address reviewer comments and improve the manuscript. However, Figures 1 and 2 are still displayed as summary histograms and have not been altered to show the spread of the individual data points. While the subsequent use of box plots is a positive adjustment, as the data is stated in the figure legends as mean and SEM this is still are not showing the spread of the data. The box plots could be altered to include the individual data points or to show mean with SD.

RESPONSE: In accordance with the reviewer#1's suggestion, the histograms in figures 2 and 3 have been changed to box plots. We have also removed the description concerning mean and SEM in figure legends. And, we added the following statements: "The horizontal line in each box indicates the median, the box shows the interquartile range (IQR), and the whiskers represent 1.5 × IQR." The descriptions in Figure legend 4, 6, 7, 8 have been changed as well.
